# Tumor microenvironment characterization in stage IV gastric cancer

**DOI:** 10.1042/BSR20201248

**Published:** 2021-01-08

**Authors:** Feng Yang, Zhenbao Wang, Xianxue Zhang

**Affiliations:** Department of Gastrointestinal Surgery, Zaozhuang Municipal Hospital, No 41, Longtouzhong Road, Shizhong District, ZaoZhuang City, Shandong Province 277100, China

**Keywords:** expression profiling, molecular subtype, Stage IV gastric cancer, tumor immunity

## Abstract

Immunotherapy is remarkably affected by the immune environment of the principal tumor. Nonetheless, the immune environment’s clinical relevance in stage IV gastric cancer (GC) is largely unknown. The gene expression profiles of 403 stage IV GC patients in the three cohorts: GEO (Gene Expression Omnibus, GSE84437 (*n*=292) and GSE62254 (*n*=77), and TCGA (The Cancer Genome Atlas, *n*=34) were used in the present study. Using four publicly available stage IV GC expression datasets, 29 immune signatures were expression profiled, and on this basis, we classified stage IV GC. The classification was conducted using the hierarchical clustering method. Three stage IV GC subtypes L, M, and H were identified representing low, medium, and high immunity, respectively. Immune correlation analysis of these three types revealed that Immune H exhibited a better prognostic outcome as well as a higher immune score compared with other subtypes. There was a noticeable difference in the three subgroups of HLA genes. Further, on comparing with other subtypes, CD86, CD80, CD274, CTLA4, PDCD1, and PDCD1LG2 had higher expression in the Immunity H subtype. In stage IV GC, potentially positive associations between immune and pathway activities were displayed, due to the enrichment of pathways including TNF signaling, Th-17 cell differentiation, and JAK-STAT signaling pathways in Immunity H vs Immunity L subtypes. External cohorts from TCGA cohort ratified these results. The identification of stage IV GC subtypes has potential clinical implications in stage IV GC treatment.

## Introduction

GC (gastric cancer), being the third most common reason for worldwide deaths due to cancer and the fifth in incidence among common cancers, exerts increasing the burden on healthcare [1,2]. PALM (Para-aortic lymph node metastasis)-characterized stage IV GC has a very poor prognosis even after isolated surgical treatment [3]. The primary approach for stage IV GC therapy remains palliative chemotherapy. The OS (overall median survival) rate of GC patients in stage IV remains at 9–11 months despite the recent advancements in chemotherapy [4]. The failure rate among several molecular targeting drugs that have entered clinical trials for GC is very high. To treat GC in a palliative setting, FDA has approved two new monoclonal antibodies: ramucirumab, which attacks VEGFR 2 (vascular endothelial growth factor receptor 2), and tratuzumab attacks HER2 (human epidermal growth factor receptor 2) [5]. Nevertheless, only few GC patients have benefited from the immune therapy. It is urgent to explore new therapeutic strategies for targeting stage IV GC patients. In the process of analysis of tumor stage in GC, and for treatment of GC, the microenvironment of tumor cells plays a vital role [6]. Markedly, the microenvironment characteristics regulating the development of stage IV GC patients have not been extensively explored.

TAMs (tumor-associated macrophages), TILs (tumor-infiltrating lymphocytes), CAFs (cancer-associated fibroblasts), BDMCs (bone marrow-derived cells), stromal cells of various types, cancer cells, and MCs (mast cells) comprise the tumor microenvironments [7]. GC advancement in patients is profoundly related to the environmental interaction of the cancer cells that finally ascertain whether the GC is eradicated, metastasized, establishes dormant micrometastases or responds to therapy and resistance [8–10]. Various cells in the GC tumor microenvironment interfered with immune surveillance, like the immune suppressor cells, Tregs (regulatory T cells), tumor-regulated microphages and MDSCs (myeloid-derived suppressive cells), and resulted in immune evasion or escape [11–13].

One of the mechanisms for escaping anticancer immune surveillance is the immune checkpoint, which is commonly witnessed among activated T cells. By inhibiting the immunosuppressive processes in several cancers (for instance, lung cancer of the non-small cell kind or metastatic melanoma), CTLA 4 and PD-1 (Cytotoxic T Lymphocyte-Associated Protein 4 and Programmed Cell Death Protein 1) enhance the immune response [14–16]. While the immunotherapy targets HER2-positive GC patients in advance stage, most GC patients do not benefit from immunotherapy [17–19]. However, the relevance (clinical) and expression profile of immune checkpoint molecules in GC has not been subjected to extensive research.

In the present study, we classified stage IV GC patients using expression profiling, in three clear subtypes: L, M, and H (Low, Medium, and High) Immunities. In the independent datasets, reproducibility and stability were demonstrated under the above-mentioned classifications. The detection of immune signature-related subtypes of stage IV GC patient may enable optimal selection of patients in stage IV GC who respond to immunotherapy.

## Materials and methods

### Stage IV GC sample datasets

The gene expression profiles and clinical data of stage IV GC were retrospectively collected from the GEO (Gene Expression Omnibus) (GSE84437 and GSE62254 https://www.ncbi.nlm.nih.gov/geo/query/acc.cgi). A total of 292 and 77 samples of Stage IV GCs (Table 1) were retrieved respectively from GSE84437 and GSE62254 using the below-mentioned rules: (a) at stage IV, (b) contained follow-up information, (c) contained gene expression profiles in GC, (d) genes with expression levels greater than 0 and more than 30% of the genes in each sample. The external validation dataset included 34 stage IV GCs which were assimilated by TCGA (The Cancer Genome Atlas; https://portal.gdc.cancer.gov/).

**Table 1:** Summary of patient demographics and characteristics

Characteristics	GSE84437 (*n*=292)	TCGA (*n*=34)	GSE62254 (*n*=77)
**Gender**			
Female	95 (32.5%)	15 (41.7%)	36 (46.8%)
Male	197 (67.5%)	21 (58.3%)	41 (53.2%)
**Age**			
<59 years	122 (41.8%)	14 (38.9%)	32 (41.6%)
≥59 years	170 (58.2%)	22 (62.1%)	45 (58.4%)
**Stage**			
I	0 (0.0%)	0 (0.0%)	0 (0.0%)
II	0 (0.0%)	0 (0.0%)	0 (0.0%)
III	0 (0.0%)	0 (0.0%)	0 (0.0%)
IV	292 (100%)	36 (100%)	77 (100%)
**Vital status**			
Living	130 (44.5%)	11 (32.3%)	31 (40.3%)
Dead	162 (55.5%)	20 (67.7%)	46 (59.7%)

### Clustering

For each stage IV GC dataset (GSE84437, GSE62254, and TCGA), the ssGSEA (single sample Gene-set Enrichment Analysis) score [20,21] was adopted to the 29 immune signatures in each stage IV GC sample to quantify the levels of enrichment. Based on these 29 ssGSEA enrichment levels of the immune signatures, we conducted hierarchical clustering of stage IV GCs.

### Tumor purity, stromal content, and the level of infiltration of immune cells

ESTIMATEA is a tool for detecting the presence of infiltrating immune cells or stromal cells in tumor tissue, as well as forecasting the purity of the tumor using gene expression data. The basis of ESTIMATEA is a ssGSEA which generates immune, stromal, and tumor scores [22]. We performed ESTIMATE to evaluate tumor purity, immune score, and stromal score) for each stage IV GC sample.

### Comparison of the proportions of the subsets of immune cells between stage IV GC subtypes

CIBERSORT [23–25] was deployed to ascertain the dimensions of subsets of 22 immune cells (human) using expression profiles. A thousand permutations were cast and *P*<0.05 was used as the basis to successfully deconvolute each sample.

### Gene-set enrichment analysis

GSEA (Gene-Set Enrichment Analysis) is a method of computation, that takes *a priori* defined set of genes and displays the concordant differences between the two biological phenotypes that are statistically significant [26,27]. GSEA was used to identify the functions and pathways in immunities H and L in the GSE84437 cohort (|logFC| > 2, FDR < 0.05).

### Statistical analysis

*P*<0.05 was considered statistically significant, and each statistical test was two-sided. The importance of survival time difference was calculated using the log-rank test and a threshold *P*-value <0.05. To compare the expression levels of checkpoint genes among the stage IV GC subtypes and to compare the immune scores, a Student’s *t* test was performed. The R software was deployed for all the statistical analyses (version 3.5.0, http://www.R-project.org).

## Results

### Expression profiling to identify three stage IV GC subtypes

**Figure 1:**
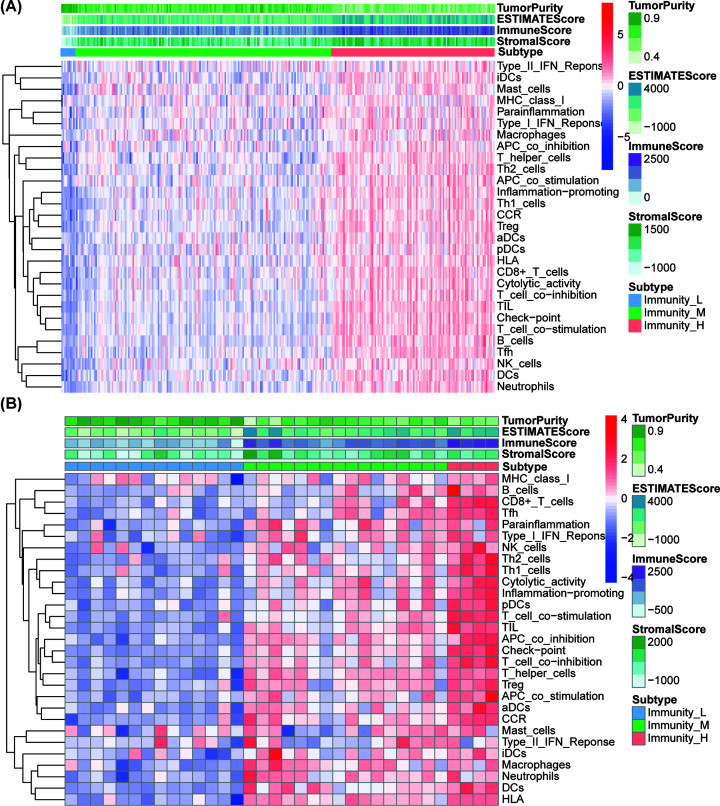
Expression profiles of the three stage IV GC subtypes in the GSE84437 and TCGA cohort Hierarchical clustering of stage IV GC yields three stable subtypes (Immunity_L, Immunity_M, and Immunity_H) in the GSE84437(**A**) and TCGA cohort (**B**). Stromal_score, immune_score, and tumor_purity were evaluated by ESTIMATE.

**Figure 2:**
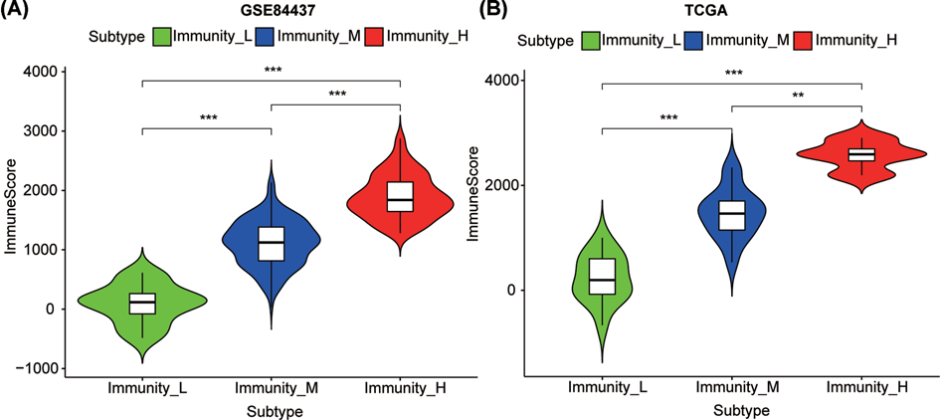
Comparison of immune_score levels between stage IV GC subtypes The immune_score levels among three stable subtypes in the GSE84437 (**A**) and TCGA cohort (**B**). **P<0.01, ***P<0.001.

Twenty-nine immune-associated gene sets that indicated different types of immune cells, pathways, and functions were analyzed (Supplementary Table S1). The ssGSEA score was used to evaluate the enrichment or activity status of immune functions, pathways, or cells in the tumor patients. Based on these 29 ssGSEA enrichment levels of the immune signatures, we conducted hierarchical clustering of stage IV GCs in GSE84437, GSE62254, and TCGA datasets, respectively. Interestingly, they had similar clustering results in which three clusters were clearly separated in the two datasets (Figure 1, Supplementary Figure S1). The three clusters were defined as immunities L, M, and H representing low, medium, and high immunities, respectively. The enrichment of lymphocyte infiltration was observed to be remarkably high in immunity_H vs. immunity_L in the two datasets. We also observed a reverse trend between the stromal score and tumor purity in the three subtypes. Tumor purity increased from immunity_H to immunity_L, however, stromal score decreased from immunity_H to immunity_L. We found that the immune scores were significantly higher in immunity_H vs. immunity_L in the GSE84437 and TCGA datasets (*P*<0.001) (Figure 2). To conclude, these results showed that immunity_L includes the highest number of tumor cells, while immunity_H includes the highest number of stromal and immune cells.

### Immune-associated genes of the three molecular subtypes

**Figure 3:**
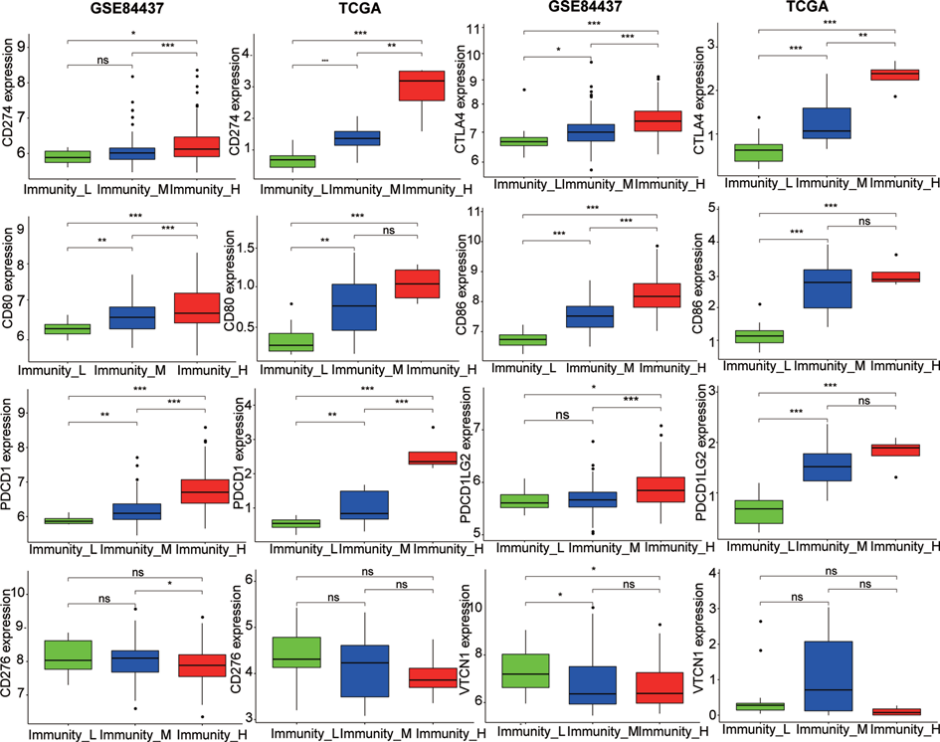
The expression of eight checkpoint molecules (PDCD1, CD274, PDCD1LG2, CTLA4, CD86, CD80, CD276, and VTCN1) in the three stage IV GC subtypes (Immunity_L, Immunity_M, and Immunity_H) *P<0.05, **P<0.01, ***P<0.001, ns: *P*>0.05.

**Figure 4:**
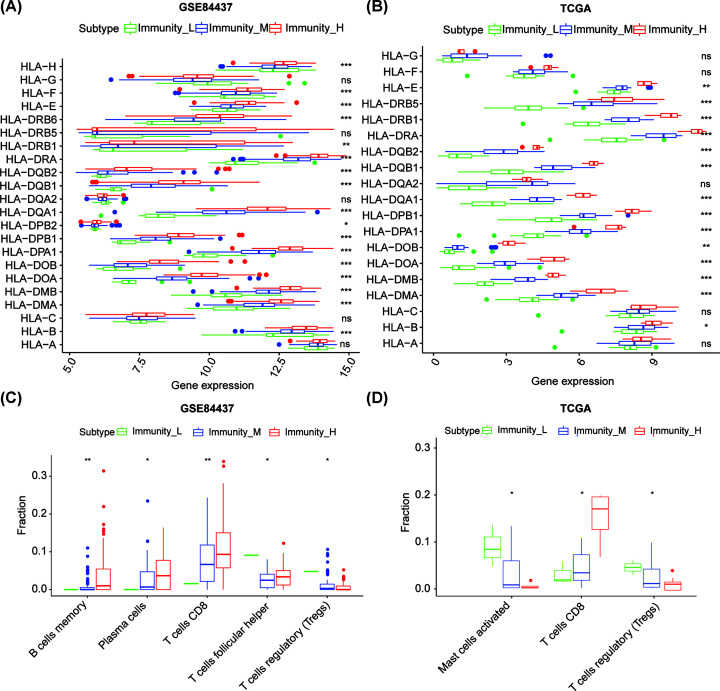
The three stage IV GC subtypes show differential phenotypes Comparison of the expression of HLA genes between stage IV GC subtypes in the GSE84437 (**A**) and TCGA cohort (**B**). Comparison of the immune cell infiltration levels between stage IV GC subtypesin the GSE84437 (**C**) and TCGA cohort (**D**). *P<0.05, ** *P*<0.01.have revised.

The blockade of immune checkpoints appears to be the most promising approach to initiate therapeutic anti-tumor immunity. The inhibitory and stimulatory pathways, that maintain self-tolerance and help with the immune response, make the immune checkpoints. To inhibit the in-built anti-tumor immune response, immune checkpoint pathways are frequently activated [28,29]. So, among the three subtypes, we analyzed the expression of eight immune checkpoints. As displayed in Figure 3, CD80, CD86, PDCD1, CTLA4, CD274, and PDCD1LG2 were more elevated in immunity_H than in other types in GSE84437. CD276 and VTCN1 levels showed no obvious difference among the three groups, and similar results were observed in the TCGA database. In the cytotoxic T cells that cause cell-mediated immunity, the MHC molecules play a significant role [30,31]. Importantly, most HLA genes displayed heightened levels of expression in immunity H than in immunity_L. HLA-A, HLA-C, HLA-G, and HLA-DQA2 did not show a significant difference among the three groups in two databases (Figure 4A,B).

### Immune cells of the three molecular subtypes

Throughout the progression of the tumor, and as a reaction to anticancer therapy, TILs can dynamically change [32,33]. The presence of lymphocytes in tumors was often associated with better clinical outcomes [34,35]. Using CIBERSORT algorithm, we first investigated the difference in 22 immune cell infiltration among three subtypes in stage IV GC (Figure 4C, Supplementary Table S2). T cells CD8 and plasma cells were in a higher proportion in immunity_H than in immunity_L in the GSE84437, whereas T cells (follicular helper), and Tregs were relatively lower (Figure 4D, *P*<0.05). CD8 T cells were found to be higher in immunity_H and MCs activated and Tregs were higher in immunity_L in the TCGA cohort.

### Prognostic significance of three molecular subtypes

**Figure 5:**
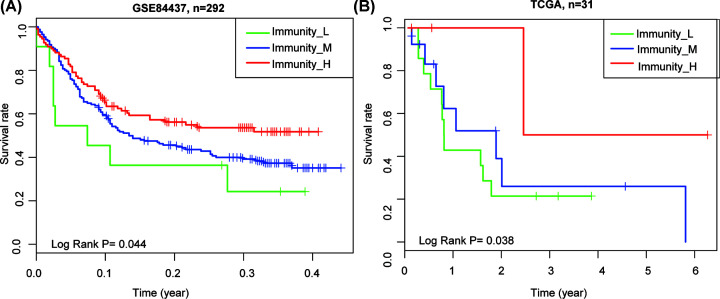
Comparison of survival prognosis between stage IV GC subtypes Survival analyses showed that these stage IV GC subtypes had distinct overall survival in the in the GSE84437 (**A**) and TCGA cohort (**B**).

The presence of a high immune tumor microenvironment usually correlated with better clinical outcomes [34,35]. Survival analyses showed that these stage IV GC subtypes had distinct overall survival in the three datasets. The prognosis for the survival of immunity_H subtype was better than that of immunities M and L subtypes, and immunity_M subtype had an improved survival prognosis than immunity_L (Figure 5A,B, Supplementary Figure S2).

### Identification of functions and pathways in immunity_H and immunity_L subtypes

**Figure 6:**
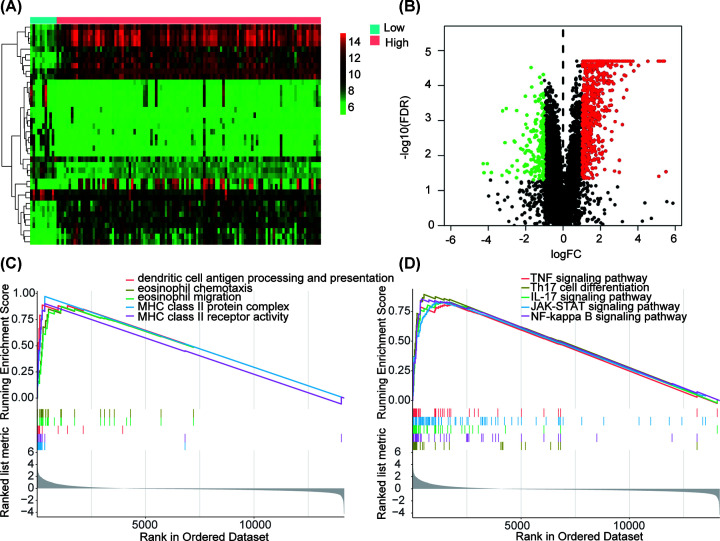
Identification of functions and pathways in Immunity_H vs. Immunity_L groups (**A**) Hierarchical clustering of the top 20 differentially expressed genes in Immunity_H vs. Immunity_L groups. (**B**) Differentially expressed genes are shown by Volcano plot. (**C**) Functions identified by GSEA in Immunity_H vs. Immunity_L groups. (**D**) GSEA identified pathways in Immunity_H vs. Immunity_L group.

A total of 21999 mRNAs were analyzed for differential expression between immunity_H and immunity_L in the GSE84437 database (Figure 6A,B). Using |Fold changes| ≥ 2 and *P*<0.05 as cutoffs, we identified 885 differentially expressed mRNAs (194 down-regulated and 691 up-regulated). Using the fold-change order of the 885 mRNAs, GSEA identified functions, and pathways in immunity_H and immunity_L groups (Supplementary Table S3). Dendritic cell antigen processing and presentation, MHC class II protein complex and receptor activity, eosinophil migration, and eosinophil chemotaxis were significantly enriched functions in immunity_H vs. immunity_L (Figure 6C,D). Th17 cell differentiation, TNF signaling pathway, IL-17 signaling pathway, the JAK-STAT signaling pathway, and NF-κB signaling pathway were obviously enriched pathways.

## Discussion

Cancer, particularly in stage IV can be seen to metastasize and spread beyond the stomach to other areas of the body. The prognosis of stage IV GC is quite poor irrespective of the use of demonstrated standard therapies like biological agents and chemotherapy [36–38].

The microenvironment of the tumor in the context of immunity is quite complex and diverse and as technology advances, so does their understanding. Various subclasses of the immune environment that affect the immune environment have been identified [39]. Treatment by manipulation of the microenvironmental factors is already showing a lot of promise in the realm of cancer treatment [40]. Prior studies have examined GC microenvironment characterization on the basis of expression profiling [41–43]. However, only a few studies have explored the microenvironment characterization of stage IV GC. To fill this study gap, we aimed at systematically analyzing heterogeneous stage IV GC microenvironment subtypes using expression profiling extracted from the GSE84437 cohort, which indicated clinical implications for stage IV GC treatment.

We found that stromal scores and immune scores decreased from immunity_H to immunity_L, while purity of tumor increased from immunity_H to immunity_L. Considering the anti-tumor immune activities, immunity_H exhibited a stronger immune cell filtration, for instance, infiltration of high levels of cytotoxic B cells and T cells. By using CIBERSORT to compute the sizes of a total of 22 immune cells in stage IV GC, we discovered that CD8 T cells tended to be noticeably higher and Tregs tended to be lower in immunity_H than in immunity_L in both datasets. The result of this analysis further confirms heightened anti-tumor immune activity in the immunity_H dataset. Compared with other subtypes, Immunity H has more favorable outcomes and this could be due to anti-tumor immune activation. Many studies have shown that TILs density has a positive association with the survival prognosis of several cancers [35,49,50].

Potential immune genes of stage IV GC were further investigated in the present study. Particularly, most HLA genes showed higher expression in immunity_H than in other types. For effective killing of tumors, CD8^+^ T cells recognize tumor peptides presented by molecules of human leukocyte antigen class I. In humans, there are three major HLA-I genes (*HLA-A, HLA-B*, and *HLA-C*) [44].

Immunity_H exhibited higher expression of *HLA-B* than other subtypes, while it showed no significant variation in *HLA-A* and *HLA-C*. The HLA-B genes may play a key role in antigen presented the stage IV GC. Stage IV GC may be provided with immune target genes by the present study. The fact that HLA-G is a crucial immunotolerance marker in cancer cell immune evasion is now largely accepted and is strongly related to progress of disease and prognosis for cancer patients [45–47]. Interestingly, we found that the HLA-G showed no significant difference in immunity_H of GC patients, which means no immunotolerance in immunity_H and better prognosis in this stage. This observation is concomitant with findings from earlier studies indicating that the expression of HLA-GC correlated with poor prognosis in GC [48]. HLA-GC showed notably higher expression levels and better prognosis in immunity_H of stage GC patients which is in line with previous studies. PDCD1, CD274, CD86, CD80, PDCD1LG2, and CTLA4 were remarkably higher in immunity_H than in other types, while CD276 and VTCN1 did not exhibit any obvious difference among the three groups. These results suggest that the immunity_H subtype with enhanced immunity may be closely associated with immune activation in stage IV GC, which may provide immunotherapy of stage IV GC using immune checkpoint blockers.

Finally, we found that MHC class II protein complex and receptor activity, toll-like receptor binding, dendritic cell antigen processing, and presentation chemokine activity were significantly enriched functions in immunity_H than in immunity_L subtype. Toll-like receptors induce dendritic cells to mature after identifying pathogen-derived products, antigen presentation, and cytokine secretion [49]. MHC class II protein complex and MHC class II receptor activity present antigen to T cells for immune recognition [50,51]. For the macrophage and lymphocyte infiltration of human cancers, chemokines are a vital determinant [52]. The results show that immunity_H has high levels of immunogenicity to antitumor. Th17 cell differentiation, TNF signaling pathway, IL-17 signaling pathway, NF-κB signaling pathway, and the JAK-STAT signaling pathways were found to be remarkably enriched pathways in immunity_H than in immunity_L subtype. JAK-STAT signaling and TNF signaling pathways are major mediators of apoptosis as well as inflammation and immunity in many cancers [53–56]. Th17 cell-derived IL-17 are dual-functioning agents acting in a cancer type [57,58]. This study revealed the positive association between immune activities and pathway activities in stage IV GC.

Currently, immunotherapy for stage IV GC is an ongoing area of investigation. Some preliminary advanced GC immunotherapy clinical trials (ATTRACTION-2, TAGS, and KEYNOTE-059) have shown significant improvement in GC patients. However, only a few people have benefited from the immune therapy. Thus, this segregation of stage IV GC may help in the categorization of stage IV GC patients who respond to immunotherapy. It is easy to conceive that patients with an immunity_H subtype of stage IV GC will have a greater likelihood of response to anti-PD-1/PD-L1 therapy when compared with patients in other stage IV GC subtypes, because PD-L1 is more noticeably demonstrated in immunity_H, and PD-L1 expression is a predictive biomarker for response to immunotherapy directed to PD-1/PD-L1 [59].

Additionally, the limitations of the present study should be pointed out. First, we lack the information on the mechanisms behind the immune score genes in stage IV GC, and experimental studies on these genes will provide important information to further enhance our understanding of their functional roles. Moreover, although we verified our findings in the Asia GC patients from public databases to the extent possible based on data availability, the immune score has not yet been tested prospectively in a clinical trial. Despite these limitations, the significant and consistent correlation of our immune score with overall survival in three independent groups indicated that it was a potentially powerful and significant immune score to evaluate the prognosis for stage IV GC patients.

## Conclusion

According to current research microenvironment, phenotypes of stage IV GC could be categorized into three molecular subtypes using expression profiles in stage IV GC. These subtypes are unique in terms of immunity characteristics, patient outcomes, and immune molecules. In addition, specific immune genes and functional pathways may direct the formation of specific phenotypes in the microenvironment. These results may go a long way in designing pathbreaking strategies for immunotherapy in stage IV GC.

## Supplementary Material

Supplementary Figures S1-S2

Supplementary Tables S1-S4

## Data Availability

The datasets analyzed for the present study can be found in the GEO (http://www.ncbi.nlm.nih.gov/geo) and TCGA (https://www.cancer.gov).

## Abbreviations

CIBERSORT: an analytical tool to provide an estimation of the abundances of member cell types in a mixed cell population, using gene expression data.
FDR: false discovery rate
GC: gastric cancer
GEO: Gene Expression Omnibus
GSEA: Gene-set Enrichment Analysis
HER2: human epidermal growth factor receptor 2
MC: mast cell
PD-L1: Programmed Cell Death-Ligand 1
PD-1: programmed cell death protein 1
ssGSEA: single sample GSEA
TIL: tumor-infiltrating lymphocyte
