# Supplementary material for: Tumor microenvironment characterization in stage IV gastric cancer
Source: Biosci Rep. 2021 Jan 8;41(1):BSR20201248. doi: 10.1042/BSR20201248 (PMC7796192; doi:10.1042/BSR20201248)
Supplement: Supplementary Figures S1-S2 [file BSR-2020-1248_supp.pdf]

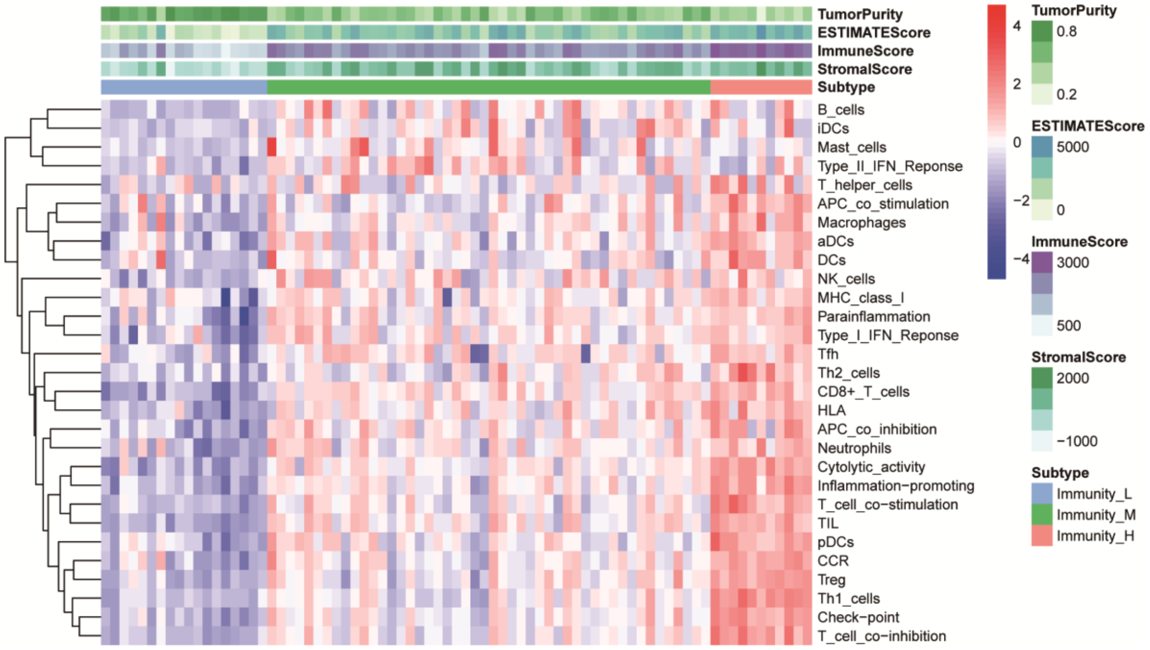

Figure S1. Hierarchical clustering of stage IV GC yields three subtypes in the Asian datasets of GSE62254

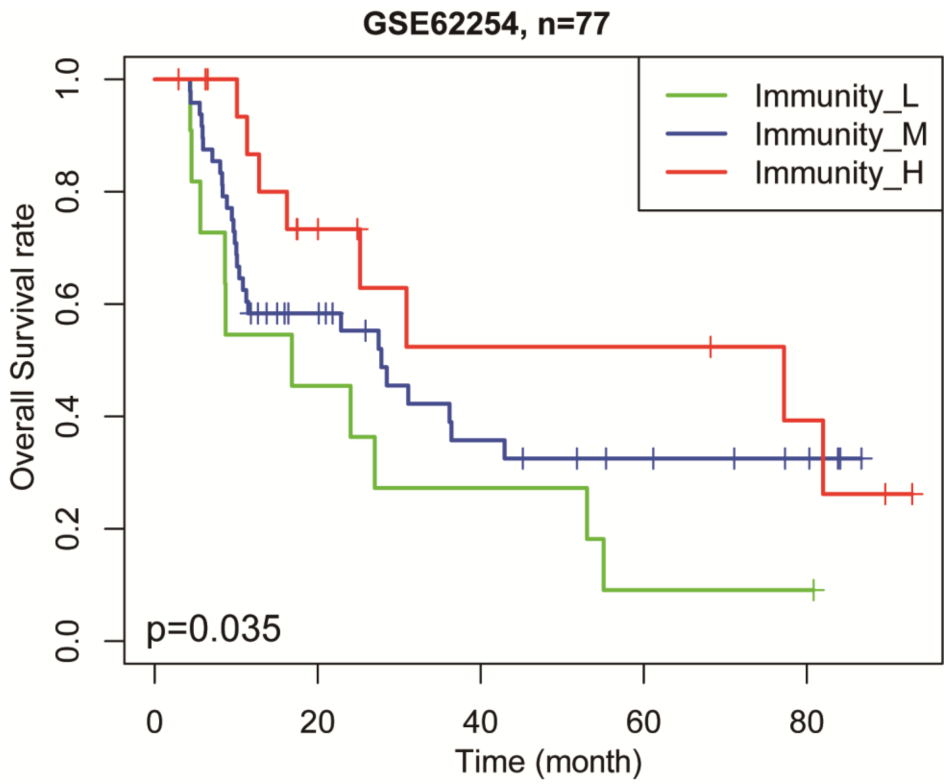

Figure S2. Comparison of survival prognosis between stage IV GC subtypes (log-rank test) in the GSE62254
